# Psychometric Examination of the Proposed Specifiers for Conduct Disorder Self-Report (PSCD) Among an Adult Community Sample From Brazil

**DOI:** 10.1177/0306624X241313282

**Published:** 2025-01-17

**Authors:** Pedro Pechorro, Bruno Bonfá-Araujo, Makilim Nunes Baptista, Cristina Nunes, Matt DeLisi, Randall T. Salekin

**Affiliations:** 1 University of Coimbra, Portugal; 2 University of Western Ontario, London, ON, Canada; 3 Pontifícia Universidade Católica de Campinas, Campinas, Brazil; 4 University of Algarve, Faro, Portugal; 5 Iowa State University, Ames, IO, USA; 6 University of Alabama, Tuscaloosa, AL, USA

**Keywords:** assessment, conduct disorder, psychopathic traits, validation

## Abstract

The Proposed Specifiers for Conduct Disorder (PSCD) is a promising novel scale that measures psychopathic traits and includes an additional conduct disorder factor that taps the antisocial dimension of psychopathy. The current study sought to broaden the application of PSCD by examining the factor structure, convergent and discriminant validity, and connections to delinquency in a young adult sample (*N* = 450; *M* = 31.91 years, *SD* = 13.02 years) obtained from the Brazilian community. Participants completed a self-report version of the PSCD along with other theoretically meaningful psychometric measures. Results supported a four-factor intercorrelated factor structure, with male participants scoring significantly higher than female participants on the PSCD total, grandiose-manipulative (GM), callous-unemotional (CU), daring-impulsive (DI), and conduct disorder (CD) factors. The four factors of the PSCD mainly presented positive, moderate to high significant inter-correlations and adequate reliability. The convergent validity with measures of the dark tetrad of personality, difficulties in emotion regulation, and self-reported delinquency also revealed positive moderate significant associations. Our findings support the use of the PSCD as a promising short, time-effective self-report measure of psychopathic traits in young adults.

## Introduction

The Proposed Specifiers for Conduct Disorder (PSCD; Salekin & Hare, 2016) is a relatively recent scale for the measurement of a broad constellation of psychopathic features among children and adolescents. Unlike a previous paradigm that primarily focused on callous and unemotional features of youth psychopathy (Frick & White, 2008), the PSCD encompasses grandiose-manipulative features that bear on the interpersonal dimension of psychopathy, callous-unemotional traits that bear on the affective deficits of psychopathy, daring-impulsive traits that bear on the lifestyle and behavioral features of psychopathy, and a recently conceptualized conduct disorder (CD) factor somewhat similar to the antisocial dimension of psychopathy (see Colins et al., 2020; Salekin, 2016(a), (b), 2017). Research regarding this recent CD factor embedded within the latent structure of the psychopathy construct and its relations to antisocial outcomes such as criminal recidivism is particularly relevant due to the scarcity of research available on this topic (see Pechorro et al., 2024).

Although a newer measure, the PSCD has already generated several validation studies spanning the self-report (Colins et al., 2023; Elhami Athar et al., 2022; López-Romero et al., 2022; Luo et al., 2021; Muratori et al., 2021; Ribeiro da Silva et al., 2022) to the parent version (Bellamy et al., 2024; Bellamy et al., 2024; López-Romero et al., 2019), utilizing diverse study samples including children ages 3 to 6 years (López-Romero et al., 2010), children ages 11 to 14 years (Bellamy et al., 2024; Muratori et al., 2021), early adolescents (Bellamy et al., 2024; López-Romero et al., 2022; Luo et al., 2021), middle adolescents (Bellamy et al., 2023; Bellamy et al., 2024; Elhami Athar et al., 2022), and middle and late adolescents (Colins et al., 2023; Ribeiro da Silva et al., 2021; Salekin et al., 2022) selected from various nations including Belgium (Colins et al., 2023), China (Luo et al., 2021), Iran (Elhami Athar et al., 2022), Italy (Muratori et al., 2021), Portugal (Ribeiro da Silva et al., 2022), Spain (López-Romero et al., 2019; López-Romero et al., 2022), and the United States (Bellamy et al., 2023; Bellamy et al., 2024; Salekin et al., 2022).

The PSCD has revealed adequate reliability and validity in diverse investigations with samples of different languages and countries. Its proposed four-factor latent model has been replicated in subsequent studies, suggesting the presence of a hierarchical or bifactor structure invariant across gender and sample type (e.g., Colins et al., 2023; Ribeiro da Silva et al., 2022). However, some studies excluded items to improve model fit (e.g., Elhami Athar et al., 2022; Luo et al., 2021; Muratori et al., 2021). Regarding reliability/internal consistency, studies have found values ranging from adequate to good when considering Cronbach’s alpha (α), McDonald’s omega (ω), and mean inter-item correlations (MIC) (e.g., Bellamy et al., 2024; Colins et al., 2023; Luo et al., 2021). The PSCD has also shown good parent-to-child agreement (Elhami Athar et al., 2023), and test-retest reliability has also proven to be more than adequate when tested (e.g., Ribeiro da Silva et al., 2022). Comparisons across gender and sample type mostly revealed the expected significantly higher scores for male and forensic participants, indicating known-group validity (e.g., Muratori et al., 2021; Ribeiro da Silva et al., 2022).

Criterion validity correlations indicate that the total and factor scores are significantly associated with numerous external criteria, including aggression, conduct problems, emotional regulation, and behavioral disorders (Bellamy et al., 2024; Colins et al., 2023; López-Romero et al., 2019; López-Romero et al., 2022; Muratori et al., 2021; Ribeiro da Silva et al., 2021; Salekin et al., 2022), school problems and educational performance (Bellamy et al., 2024; Colins et al., 2023; Elhami Athar et al., 2022), and various externalizing features spanning substance use, rule-breaking behavior, aggressive behavior, proactive aggression, and reactive aggression (Bellamy et al., 2024; Colins et al., 2023; Elhami Athar et al., 2022; López-Romero et al., 2022; Luo et al., 2021; Ribeiro da Silva et al., 2022; Salekin et al., 2022).

In addition to these findings, prior research has also shown that the PSCD is significantly correlated with other psychopathy measures, including the Youth Psychopathic Traits Inventory (YPI), Antisocial Process Screening Device (APSD), Inventory of Callous Unemotional Traits (ICU) (Bellamy et al., 2023; Luo et al., 2021) and the Strengths and Difficulties Questionnaire which spans psychopathology, behavior problems, and prosocial behaviors (López-Romero et al., 2022; Muratori et al., 2021). These initial findings support the broadband associations between psychopathic features and myriad behavioral problems, delinquency, and justice system involvement, evidenced by ample prior research studies (e.g., DeLisi, 2016; Hare, 1999; Lee & Kim, 2022; Ray et al., 2020). Nevertheless, this measure remains unavailable in some countries, including Brazil. Previous studies have demonstrated that psychopathy in Brazil exhibits similar characteristics to those found in other countries. In addition, research indicates that individuals with psychopathic tendencies in Brazil show higher levels of transgressions, risk-taking, deceitfulness, and callousness than in some other countries (Machado et al., 2023). Additionally, there is a positive association between psychopathy and the Brazilian concept of “Jeitinho” (i.e., strategies to overcome specific problems; Machado et al., 2024).

### Current Study

With the development of a new measure of psychopathic features, validation studies employing data from samples from diverse ethnic and national backgrounds are critical. In this vein, the current study sought to broaden the application of PSCD in multiple ways by using young adults obtained from the Brazilian community. We are unaware of any previous investigations examining whether the PSCD performs adequately in young adults as it does in children and youth. This step could help further understand whether the measure can be extended to this age band. However, it is essential to examine psychopathic trait measures that were originally developed for children and adolescents among such adult samples especially since CD is now considered a life course disorder (e.g., Colins & Andershed, 2016). Furthermore, to date, no studies of the PSCD’s psychometric properties have been conducted in Brazil. In this regard, the current study is important scientifically in terms of measurement validation and practically in terms of evaluating a measure of a psychological condition that is very costly to society (DeLisi, 2016; Hare, 1999; Reidy et al., 2015).

We hypothesized that the PSCD would display the expected four-factor latent structure, that the four factors of the PSCD would be significantly correlated, display satisfactory internal consistency/reliability, and demonstrate significant convergent validity with measures of dark traits of personality, difficulties in emotion regulation, and show a positive relation with delinquency. Concerning the PSCD and dark traits, we expected the subscales to correlate highest with their closest corresponding scale. We also expected that male participants would score significantly higher than female participants on the four factors of the PSCD and total score.

## Method

### Participants

Our sample consisted of 450 Brazilians, with a mean age of 32 years (*SD* = 13.02 years; range = 18–70 years), subdivided into males (*N* = 206; *M* = 31.19 years; *SD* = 12.60 years; range = 18–70 years) and females (*N* = 244; M = 32.52 years; *SD* = 13.35 years; range = 18–70 years). The majority of the participants were single (69.6%) or married (20%), mainly from the Southeast (48.9%) and South (21.3%) regions of Brazil, living in large and medium-sized cities (84.2%). Regarding employment status, most participants were full-time public or private sector workers (36.3%), while some were unemployed (22%). The reported predominant monthly income was from one to three minimum national wages (41.1%; from US$ 240 to US$ 720 per month) and less than one minimum national wage (29.7%; approximately US$240 per month). No differences were found between males and females regarding age (*F* = 1.15, *p* = .28), marital status (χ^2^ = 7.02, *p* = .06), city size (χ^2^ = 1.58, *p* = .67), employment status (χ^2^ = 8.80, *p* = .12), monthly income (χ^2^ = 8.23, *p* = .14). However, gender differences were found in terms of region of origin (χ^2^ = 11.84, *p* = .02), with a greater proportion of the South region of Brazil participating in the current study.

### Measures

#### Sociodemographic Questionnaire

Participants were asked to share personal information such as sex, age, civil status, city size, labor information, and monthly income.

#### Proposed Specifiers for Conduct Disorder (PSCD; Salekin & Hare, 2016)

The PSCD was developed as a measure of the broader construct of psychopathy, initially focusing on assessing children and adolescents. In addition to Conduct Disorder (CD) (e.g., “19. I have stolen things”), the PSCD assesses interpersonal (Grandiose-Manipulative, e.g., “5. I take advantage of others”), affective (Callous-Unemotional, e.g., “12. I rarely feel guilty or remorse”), and lifestyle (Daring-Impulsive, e.g., “16. I feel like I need a lot of stimulation”) traits of the psychopathic personality. The PSCD has 24 items (six for each factor) answered on a 3-point Likert-type scale (0 = *Not true*, 1 = *Somewhat true*, and 2 = *True*). Items are summed to obtain factor scores and total score. Higher scores indicate higher levels of psychopathic-like traits. The PSCD has been validated in several countries, and the reliability coefficients alpha (α) are usually adequate, ranging from α = .80 (Luo et al., 2021) to α = .93 (Bellamy et al., 2024; Ribeiro da Silva et al., 2022). The current study used the self-report version of the PSCD. Reliability values for the current study are presented in the Results section.

#### Short Dark Tetrad (SD4; Paulhus et al., 2021)

The SD4 is a scale designed to assess the Dark Tetrad of personality, namely narcissism-special (e.g., “8. People see me as a natural leader”), Machiavellianism-crafty (e.g., “1. It’s not wise to let people know your secrets”), psychopathy-wild (e.g., “15. People often say I’m out of control”), and sadism-mean (e.g., “22. Watching a fist-fight excites me”). The SD4 scale has 28 items, 7 for each dimension, answered on a 5-point Likert scale (1 = *Strongly Disagree* to 5 = *Strongly Agree*). Items are summed to obtain factor scores; as usually recommended, an SD4 total score was not calculated. Higher scores indicate higher levels of dark personality characteristics. The original study reported α reliability coefficients that ranged from .78 to .83. This instrument showed adequate psychometric properties in Portuguese (Guilhermino et al., 2024; Pechorro et al., 2023). In the present study, we mainly found fair reliability for narcissism (α = .72, ω = .73), Machiavellianism (α = .73, ω = .74), psychopathy (α = .80, ω = .81), and sadism (α = .83, ω = .83).

#### Difficulties in Emotion Regulation Scale (DERS; Gratz & Roemer, 2004)

The DERS is an instrument that assesses emotional dysregulation through 6 factors, namely, non-acceptance of emotional responses (e.g., “11. When I’m upset, I become angry with myself for feeling that way”), difficulty engaging in goal-directed behavior (e.g., “13. When I’m upset, I have difficulty getting work done”), impulse control difficulties (e.g., “3. I experience my emotions as overwhelming and out of control”), lack of emotional awareness (e.g., “2. I pay attention to how I feel”), limited access to emotion regulation strategies (e.g., “15. When I’m upset, I believe that I will remain that way for a long time”), and lack of emotional clarity (e.g., “4. I have no idea how I am feeling”). The instrument uses a 5-point Likert scale (1 = *Almost never* to 5 = *Always*). Items are summed to obtain factor scores and total score. Higher scores indicate higher levels of difficulties in emotion regulation characteristics. In its original study, the DERS showed adequate internal consistency, varying from .80 to .85 (Gratz & Roemer, 2004). This instrument showed adequate psychometric properties in Brazilian Portuguese (Miguel et al., 2017). In the present study, we mainly found fair and good reliability for non-acceptance of emotional responses (α = .71, ω = .72), difficulty engaging in goal-directed behavior (α = .84, ω = .85), impulse control difficulties (α = .80, ω = .82), lack of emotional awareness (α = .77, ω = .79), limited access to emotion regulation strategies (α = .86, ω = .88), lack of emotional clarity (α = .87, ω = .88), and DERS total (α = .85, ω = .87).

#### Add Health Self-Report Delinquency (AHSRD; Harris, 2013)

The AHSRD assesses delinquency behavior by considering nonviolent and violent delinquency. The instrument has 17 items (e.g., “3. Take something from a store without paying for it?”—nonviolent, and “15. Pull a knife or a gun on someone?”—violent). The instrument uses a 4-point Likert-type scale (0 = *None* to 3 = *Five or more times*). Items are summed to obtain factor scores and total score. Higher scores indicate higher levels of delinquency. The AHSRD showed excellent internal consistency in previous studies ( Pechorro et al., 2019 ). In the present study, we mainly found fair and good reliability for nonviolent delinquency (α = .83, ω = .84), violent delinquency (α = .86, ω = .87), and AHSRD total (α = .90, ω = .91).

### Procedure

#### Translation of the PSCD

We followed the recommendations made by the International Test Commission (2017), which consisted of the following steps for proper translation, including: translation, evaluation, back-translation, presentation to the original authors, and pilot study. Two expert judges with knowledge in psychology and psychological assessment, proficient in English and Brazilian Portuguese, were invited to translate the original items. They aimed not only to translate the items but also to consider the particularities of the Brazilian Portuguese language. The authors later synthesized the translations into a single file and assessed by two PhD students proficient in both languages, who also performed the back-translation. Following back-translation, the instrument was sent to the original author for approval (RT Salekin). We carefully considered all the comments provided by the original author.

We then submitted the project to an Ethical Board according to the Declaration of Helsinki. After approval was obtained from the University São Francisco (São Paulo, Brazil), all instruments were allocated on Google Forms. We used a link posted on the research group’s social media pages to recruit participants. Individuals recruited were instructed that participants needed to be 18 years or older, and no monetary compensation was offered (i.e., voluntary participation). Furthermore, participants were instructed about the project’s confidentiality and anonymity. Following participant consent, each participant completed a battery of measures using an online platform. The online platform and measurement process followed the same order outlined in the instruments section. The survey took approximately 20 min to complete.

### Data Analysis

We first performed descriptive statistics for the PSCD with SPSS v29 (IBM Corp, 2022). Next, all confirmatory factor analysis (CFA) models were conducted on RStudio with the lavaan package (Rosseel, 2012). To examine all possible models previously proposed, that is, unidimensional, four-factor inter-correlated, and four-factor second order, we used the Diagonal Weighted Least Squares (DWLS) estimator and relied on commonly used fit indexes (Brown, 2015; Maroco, 2024): Chi-square/degrees of freedom (χ^2^/df), Comparative Fit Index (CFI), Tucker Lewis Index (TLI), and Root Mean Square Error of Approximation (RMSEA), with the usual reference values (below 5 for chi-square/degrees of freedom, above .90 for CFI and TLI, below .08 for RMSEA). Measurement invariance was examined using ΔCFI, ΔRMSEA, and Δχ^2^(df) (Putnick & Bornstein, 2016; Van De Schoot et al., 2012 ), we considered non-invariance if ΔCFI was higher than .01, and ΔRMSEA was higher than .015 (Chen, 2007). Measurement equivalence is an important prerequisite for the investigation of mean differences, but is often neglected for psychopathy instruments.

We estimated the reliability/internal consistency of the psychometric measures using Cronbach’s alpha (α), McDonald’s omega (ω; both if ≤.59 low, ≥.60 marginal, ≥.70 fair/acceptable, ≥.80 good, and ≥.90 excellent), and mean inter-item correlations (adequate if between .15 and .50; Cicchetti, 1994; Clark & Watson, 2016, 2019; Deng & Chan, 2017; Maroco, 2021). We examined the associations between measures using Pearson product-moment correlation coefficients and mean comparisons between males and females using ANOVAs, including power and effect size (Maroco, 2021).

## Results

Table 1 presents the descriptive information for the PSCD items considering the total sample. Skewness and kurtosis were mostly between the recommended −2 and 2 range, which is indicative of approximately normal distributions.

**Table 1.** Descriptive Statistics for the PSCD.

Items	Minimum	Maximum	Mean	Std. Deviation	Skewness	Kurtosis
PSCD 1	0	2	.74	.68	.37	–.84
PSCD 2	0	2	.46	.63	1.06	.02
PSCD 3	0	2	.99	.71	.01	–1.03
PSCD 4	0	2	.78	.82	.41	–1.40
PSCD 5	0	2	.33	.58	1.60	1.49
PSCD 6	0	2	1.07	.75	–.11	–1.20
PSCD 7	0	2	.38	.65	1.48	.87
PSCD 8	0	2	1.01	.82	–.01	–1.51
PSCD 9	0	2	.47	.68	1.14	–.01
PSCD 10	0	2	.50	.74	1.09	–.34
PSCD 11	0	2	.37	.66	1.53	.95
PSCD 12	0	2	.40	.68	1.41	.56
PSCD 13	0	2	.73	.71	.44	–.92
PSCD 14	0	2	.83	.77	.31	–1.27
PSCD 15	0	2	.69	.78	.59	–1.12
PSCD 16	0	2	1.10	.82	–.18	–1.51
PSCD 17	0	2	1.03	.75	–.05	–1.21
PSCD 18	0	2	.49	.71	1.10	–.19
PSCD 19	0	2	.59	.81	.87	–.92
PSCD 20	0	2	.51	.77	1.08	–.44
PSCD 21	0	2	.27	.61	2.12	3.01
PSCD 22	0	2	.33	.61	1.68	1.64
PSCD 23	0	2	.37	.69	1.70	1.28
PSCD 24	0	2	1.22	.77	–.37	–1.23

*Note*. PSCD = Proposed Specifiers for Conduct Disorder.

Subsequently, we tested three confirmatory models per sample, that is, the total sample, the male sample, and the female sample (see Table 2). The 4-factor model inter-correlated model provided the best fit when considering the three samples.

**Table 2.** Goodness of Fit for the Different Models of the PSCD.

Models	χ^2^(df)	CFI	TLI	RMSEA (90% C.I.)
Total sample				
1-factor	1065.03/252	.878	.866	.085(.080, .090)
** 4-factor**	**598.23/246**	**.947**	**.941**	**.056(.051, .062)**
4-factor 2nd order	647.76/248	.940	.933	.060(.054, .066)
Male sample				
1-factor	673.83/252	.871	.859	.090(.082, .099)
** 4-factor**	**417.02/246**	**.948**	**.942**	**.058(.048, .068)**
4-factor 2^nd^ order	433.45/248	.944	.937	.060(.051, .070)
Female sample				
1-factor	597.18/252	.868	.856	.075(.067, .083)
** 4-factor**	**365.86/246**	**.954**	**.949**	**.045(.035, .054)**
4-factor 2^nd^ order	410.84/248	.938	.931	.052(.043, .061)

*Notes*. PSCD = Proposed Specifiers for Conduct Disorder.

Presented in Table 3 are the standardized loadings for the 4-factor inter-correlated robust model of the PSCD. Loading values mainly were above the recommended .30 cut-off, except for two items from the GM factor, namely item 2. I am a very important person, and item 3. I am very good at most things I do.

**Table 3.** Loadings for the Confirmatory 4-factor Inter-correlated Model Structure of the PSCD.

Items	Loadings
Grandiose-Manipulative (GM)	
1. I can turn on the charm in any situation.	.578
2. I am a very important person.	*.132*
3. I am very good at most things I do.	*.196*
4. Lying is easy for me.	.666
5. I take advantage of others.	.665
6. I am a natural storyteller.	.610
Callous-Unemotional (CU)	
7. I don’t think about how I hurt others.	.515
8. I can turn and walk away from someone who is hurt.	.541
9. I do not seem to care if people are happy or upset.	.576
10. I like it when others are afraid of me.	.758
11. Some people consider me to be a mean person.	.678
12. I rarely feel guilt or remorse.	.616
Daring-Impulsive (DI)	
13. I am daring.	.629
14. I like a lot of change and adventure.	.576
15. I get a thrill out of doing risky things.	.799
16. I feel like I need a lot of stimulation.	.520
17. I like to live in the moment.	.567
18. Some people think I am reckless.	.715
Conduct Disorder (CD)	
19. I have stolen things.	.548
20. I have engaged in physical aggression against animals or people.	.509
21. I have destroyed property.	.514
22. I break (violate) a lot of rules.	.699
23. I started breaking rules before the age of 10 years.	.584
24. I can be argumentative and defiant. [oppositional]	.615

*Note*. PSCD = Proposed Specifiers for Conduct Disorder.

Next, we examined configural, metric, and scalar measurement invariance across genders considering the 4-factor model inter-correlated model and the 4-factor 2nd order model (see Table 4). Cross-gender measurement invariance was fully supported.

**Table 4.** Tests for Invariance of the 4-factor and 4-factor 2nd Order Models of the PSCD.

4-factor intercorrelated model	χ^2^(df)	Δχ^2^(df)	CFI	ΔCFI	RMSEA	ΔRMSEA
Configural invariance	1030.6 (492)	—	.951	—	.070	—
Metric invariance	1113.6 (512)	28.21 (20)	.945	–.006	.072	.003
Scalar invariance	1115.3 (532)	4.62 (20)	.947	.002	.070	–.002
4-factor 2nd order model	χ^2^(df)	Δχ^2^(df)	CFI	ΔCFI	RMSEA	ΔRMSEA
Configural invariance	1124.7 (496)	—	.943	—	.075	—
Metric invariance	1211.0 (519)	26.26 (23)	.937	–.006	.077	.002
Scalar invariance	1212.3 (538)	3.41 (19)	.939	.002	.075	–.002

*Note*. χ^2^(df) = chi-square (degrees of freedom); CFI = Comparative Fit Index; RMSEA = Root Mean Square Error of Approximation.

Table 5 displays the correlation matrix and the reliability/internal consistency of the PSCD and its four factors. The correlations were positive and statistically significant, varying from moderate to strong, as expected. The reliability/internal consistency values mostly varied from adequate to good. The exception was the GM factor that exhibited slightly lower alpha and omega coefficients (below .70) than that of the CU, DI, and CD subscales, although these estimates are still reasonable given the very few items per scale.

**Table 5.** Correlation Matrix and Reliability for the PSCD and Its Factors.

	PSCD total	GM	CU	DI	CD
PSCD total	1				
GM	.71***	1			
CU	.72***	.41***	1		
DI	.78***	.44***	.31***	1	
CD	.77***	.36***	.41***	.53***	1
Alpha	.87	.65	.79	.80	.78
Omega	.88	.66	.80	.81	.79
MIC	.22	.24	.39	.40	.37

*Note*. PSCD = Proposed Specifiers for Conduct Disorder; GM = Grandiose-Manipulative; CU = Callous-Unemotional; DI = Daring-Impulsive; CD = Conduct Disorder; Alpha = Cronbach’s Alpha; Omega = McDonald’s Omega; MIC = mean inter-item correlation.

*** *p* ≤ .001 level.

To examine the convergent validity of the PSCD, we tested the correlation coefficients between PSCD scores and the SD4, the DERS, and the AHSRD (see Table 6). We found positive significant correlations with all pathological traits measured by the SD4; the highest correlation was between the PSCD total score and the psychopathy factor of the SD4, while the lowest correlation was between the CD factor of the PSCD and the narcissism factor of the SD4 (see Table 6, panel A). Regarding emotional dysregulation measured by the DERS, we found a very wide range of correlations, positive and negative, significant and non-significant; the highest correlation was between the CD factor of the PSCD and the ICD factor of the DERS, while the lowest correlation coefficient was between the GM factor of the PSCD and the LEA factor of the DERS. It is important to mention that the DI and CD factors of the PSCD mostly showed problematic correlation coefficients with the DERS factors, and the GM and CU traits scales showed fewer difficulties, although all four factors showed meaningful associations with the DERS (see Table 6, panel B). In terms of delinquency, we found positive significant correlation coefficients with both violent and nonviolent self-reported delinquency as measured by the AHSRD; the highest correlations were between the PSCD total scores and all forms of delinquency, while the lowest correlation coefficient was between the GM factor of the PSCD and the non-violent factor of the AHSRD (see Table 6, panel C).

**Table 6.** Convergent Validity of the PSCD With SD4, DERS, and AHSRD Measures.

	PSCD total	GM	CU	DI	CD
Panel A					
SD4 Mach	.55***	.63***	.29***	.42***	.32***
SD4 Narc	.50***	.46***	.43***	.33***	.28***
SD4 Psyc	.63***	.33***	.38***	.56***	.56***
SD4 Sadi	.62***	.39***	.47***	.45***	.52***
Panel B					
DERS NER	.18*	.16*	.08	.12*	.14*
DERS LEC	.20*	.05	.13*	.18*	.22**
DERS LEARS	.29***	.08	.14*	.34***	.31***
DERS LEA	–.04	–.08	.05	–.05	.07
DERS DEGB	.15*	.05	–.03	.22*	.22*
DERS ICD	.32***	.07	.07	.33***	.37***
DERS total	.31***	.09	.14*	.26**	.32***
Panel C					
AHSRD NV	.39***	.20*	.21*	.33***	.27**
AHSRD V	.44***	.23*	.27**	.34***	.38***
AHSRD total	.45***	.24**	.27**	.38***	.36***

*Note*. PSCD = Proposed Specifiers for Conduct Disorder; SD4 = Short Dark Tetrad; DERS = Difficulties in Emotion Regulation Scale; AHSRD = Add Health Self-Report Delinquency; GM = Grandiose-Manipulative; CU = Callous-Unemotional; DI = Daring-Impulsive; CD = Conduct Disorder; Mach = Machiavellianism; Narc = Narcissism; Psyc = Psychopathy; Sadi = Sadism; NER = Non-acceptance of emotional response; DEGB = Difficulty engaging in goal-directed behavior; ICD = Impulse control difficulties; LEA = Lack of emotional awareness; LEARS = Limited access to emotion regulation strategies; LEC = Lack of emotional clarity; NV = Nonviolent; V = Violent.

*** *p* ≤ .001 level, **p ≤ .01 level, * *p* ≤ .05 level.

Lastly, we performed ANOVAs to compare the male and female participants in terms of known-groups validity (see Table 7). As expected, male participants presented significantly higher results for all factors of the PSCD and the total score.

**Table 7.** Known-groups Validity for the PSCD and Its Factors.

Factors	Males *M* (*SD*)	Females *M* (*SD*)	*F* (*p-*value)	Effect size η_*p*_^2^ (power)
PSCD total	18.42 (9.09)	13.29 (7.66)	42.26 (<.001)	.09 (1)
Grandiose-manipulative	4.78 (2.65)	4.04 (2.41)	9.70 (=.002)	.02 (.88)
Callous-unemotional	3.97 (3.11)	2.44 (2.68)	31.84 (<.001)	.07 (1)
Daring-impulsive	5.44 (3.36)	4.39 (3.01)	12.03 (=.001)	.03 (.93)
Conduct disorder	4.23 (3.27)	2.43 (2.30)	46.96 (<.001)	.10 (1)

*Note*. PSCD = Proposed Specifiers for Conduct Disorder; *M* = Mean; *SD* = Standard Deviation; η_*p*_^2^ = partial eta squared.

## Discussion

The PSCD is a relatively new measure for the examination of psychopathic traits and conduct disorder. It has been receiving encouraging results from a variety of countries which suggests that it warrants further investigation (e.g., Luo et al., 2021). The current study aimed to broaden the application of the PSCD by using young adults selected from the Brazilian community. To date, there are no studies on this measure with an emerging adult sample, and no studies have been conducted in Brazil. The results obtained by us in this current investigation suggest that the PSCD is a useful psychometric measure for assessing psychopathic traits in young adulthood in line with a four-factor model of psychopathy (e.g., Williams et al., 2007). The results show that the PSCD is linked with deficits in emotional functioning, goal-directed behavior, regulatory problems, and total, nonviolent, and violent delinquency. We elaborate on the findings below.

We examined the factor structure of the PSCD using CFA. Findings revealed that the correlated four-factor was the best-fitting model for the overall sample, men and women. This model received good fit statistics and indicates that the structure for the condition is best represented as a disorder but also one that has four intercorrelated components, namely grandiose manipulative (GM) traits, callous-unemotional (CU) traits, daring impulsive (DI) traits, and conduct disorder (CD). The findings indicate that conduct disorder can be conceptualized with psychopathic personality traits and that models that incorporate only one component of psychopathy, such as the CU traits-based model, are counter-indicated. Similarly, arguments to remove antisocial characteristics, at least with respect to conduct disorder, do not appear to be warranted by the results presented in this and other studies (e.g., Hare et al., 2018). Two items did not perform as well as expected in the current sample, including “I am a very important person” and “I am very good at most things I do.” Some preliminary work has demonstrated that slight alterations to these items to indicate being “better” than others can improve the fit (“I am better than others at most things I do”), although additional work is still needed given that these findings may be sample specific. Overall, the correlated 4-factor model fits the data very well, but the 4-factor 2nd-order model also presented good results.

In terms of measurement invariance, our findings showed that the measure was invariant across gender groups when considering the two best-fitting models, namely the 4-factor correlated model and the 4-factor 2nd-order model. Measurement equivalence is an important prerequisite for the investigation of mean differences, and research on gender differences in psychopathy indicates that men generally exhibit higher psychopathy scores than women. Confirming previous studies (e.g., Bellamy et al., 2024; Ribeiro da Silva et al., 2022), our findings did demonstrate the presence of cross-gender invariance.

The reliability of the PSCD was very good at the total score level. The subscales mostly exhibited good homogeneity, although regarding the GM subscale, the alpha and omega coefficient estimates were somewhat lower (.65 and .66, respectively). Using MIC values, the reliability estimates were estimated to be good regarding all the subscales. Taken together, these estimates suggest that the internal consistency for the PSCD was strong, providing further support for the PSCD’s broad set of symptoms and components, indicating that they cohere in a meaningful way and with key theoretical models (Cleckley, 1976; Hare, 2003).

The convergent validity of the PSCD was examined primarily with the dark tetrad (SD4). Concerning convergent validity, the PSCD total score exhibited its highest correlation coefficient with the SD4 psychopathy scale. It also exhibited moderate to high correlation coefficients with the SD4 dark tetrad subscales. The PSCD components (GM, CU, DI) also exhibited meaningful relations with their highest correlation coefficients being with their closest corresponding scales demonstrating good convergent-discriminant validity at the subscale level. Specifically, the GM scale of the PSCD had the highest correlation coefficients with the SD4 Machiavellianism and the SD4 narcissism scales which fit with confidence, grandiosity, and manipulation measured by the PSCD GM scale. The CU scale of the PSCD had its highest correlation coefficient with the SD4 sadism subscale, which corresponds with the lack of remorse and empathy that is captured with the PSCD CU scale. The PSCD DI component exhibited its highest correlation coefficients with the psychopathy scale, although there is no parallel scale for the PSCD DI scale on the SD4. Therefore, these findings indicate that the PSCD demonstrates good convergent validity at the full-scale level and good convergent-discriminant validity at the subscale level.

We tested the relation between the PSCD and regulatory functioning pertaining to emotion processing, goal-directed behavior, and impulsivity. These relations and correlation coefficients were examined primarily between the PSCD and the DERS subscales. The findings indicated that the PSCD was positively related to several emotional deficits including non-acceptance of emotional response, lack of emotional clarity, and limited access to emotional regulation strategies, all of which indicate significant problems in emotional processing and a lower capacity to differentiate aversive events from feelings (Gratz & Roemer, 2004). In addition, those with elevated PSCD scores exhibited difficulty engaging in self-directed behavior and higher impulsivity levels. These findings show support for the PSCD as a psychopathic traits measure indicating that those with psychopathic traits show less emotional understanding of others and less sharing of emotions. These findings also suggest that those with elevated psychopathic traits are less goal-directed and exhibit higher levels of impulsivity. This may be due to their shallow affect, lack of emotion, and reward-seeking behavior, as has been underscored in theoretical writings related to the psychopathic personality (Cleckley, 1976; Hare, 2003) and research (Harpur et al., 1989). As previously demonstrated, psychopathy is positively associated with emotional dysregulation as measured by the DERS (Garofalo et al., 2020) in both American and Italian samples, including general community members and offenders (Garofalo et al., 2018).

Regarding the components, it should be noted that the findings differ when examining the individual psychopathy components. For instance, individuals with elevated GM and CU traits did not exhibit difficulty engaging in goal-directed behavior and did not exhibit problems with impulsivity, whereas those with elevated DI and CD traits did exhibit these difficulties. Interestingly, those with elevated GM traits also did not exhibit a lack of emotional clarity and did not exhibit limited access to emotion regulation strategies. In contrast, those with elevated CU, DI, and CD did exhibit some of these deficits. Those with GM traits did, however, exhibit other emotion-based deficits such as non-acceptance of an emotional response. These findings further support the PSCD as a measure of psychopathic traits as these findings fit with theory and past research on psychopathy and the PSCD (e.g., Harpur et al., 1989; Salekin et al., 2022). Moreover, they highlight that component-level analyses can be helpful in understanding the clinical differences in individuals with (various) elevated psychopathic traits.

We examined the PSCD’s relation with self-reported delinquency. The findings from the current investigation demonstrated that the PSCD scores were related to total counts for delinquency. This finding is consistent with past research and theory which has shown that psychopathy is correlated with delinquency and crime (DeLisi, 2016; Geerings et al., 2021; Leistico et al., 2008). Further, the correlation coefficients were rather strong in magnitude indicating that the PSCD is potentially more concerningly related to self-reported delinquency than perhaps other related disorders alone. The findings from the current study also indicated that elevated PSCD scores were linked with both violent and nonviolent offending further fitting with other research on this topic ( Geerlings et al., 2020 ; Leistico et al., 2008; Mendez et al., 2023). These findings also correspond with Hare’s (2003) notion that those with elevated psychopathic traits demonstrate versatility in their criminal behavior and this may include destruction of property, theft, as well as crimes of violence against others.

### Limitations

Our findings must be interpreted in the context of some limitations. Specifically, the study had a medium-sized sample drawn from an adult population and future studies should consider larger samples. Second, we focused on adults, and future research in Brazil (and other countries) should focus on other age categories (children and adolescents). Third, the current study utilized self-report data, and thus, future studies should utilize multi-source data to reduce method variance and examine the constructs from various vantage points. Fourth, the current study did not include longitudinal data, and we, therefore, could not examine future predictive outcomes. It will be important for future research to examine the PSCD, its stability, and the predictive ability of prospective behaviors.

### Implications and Conclusion

The validation of the PSCD in a Brazilian sample provides evidence for its cross-cultural pertinency, supporting further studies in varied populations. The four-factor model (i.e., GM, CU, DI, CD) provides a framework for prospect research on these traits’ interaction and individual contributions. Future studies should also explore these traits in Brazilian children and adolescents to understand their developmental trajectory. Clinicians can use the PSCD to identify people at risk for severe emotional and behavioral problems.

The findings from the current study indicate that the PSCD holds promise as a self-report measure for assessing psychopathy in Brazil. These findings support and greatly extend past research showing the PSCD to produce reliable and valid results. The current study demonstrated that the PSCD was related to an alternate psychopathy scale with additional dark tetrad traits, emotion-related difficulties, and other regulatory problems. In addition, the present study showed that the PSCD was related to both violent and nonviolent delinquency. Although positive findings concerning psychometric properties, further replication and expansion of these study findings are required, especially if we are to learn more about the psychopathic personality and its relation to CD. Such research may eventually indicate that the PSCD allows for the assessment of psychopathic traits in combination with CD and can further facilitate our understanding of those with antisocial traits across different age bands and different countries. This should facilitate better assessment, characterization, and etiological understanding of those with antisocial traits. In addition, it should facilitate the development of tailored interventions, which could be very important across various life stages.

## Data Availability

The dataset analyzed during the current study is available from the corresponding author upon reasonable request.
